# Bone marrow-derived mesenchymal stem cells improve cognitive impairment in an Alzheimer’s disease model by increasing the expression of microRNA-146a in hippocampus

**DOI:** 10.1038/s41598-020-67460-1

**Published:** 2020-07-01

**Authors:** Masako Nakano, Kenta Kubota, Eiji Kobayashi, Takako S. Chikenji, Yuki Saito, Naoto Konari, Mineko Fujimiya

**Affiliations:** 1 Department of Anatomy, Sapporo Medical University School of Medicine, Sapporo, Hokkaido Japan; 2 Department of Physical Therapy, Hokkaido Chitose Rehabilitation College, Chitose, Hokkaido Japan; 3 Graduate School of Rehabilitation Science, Hokkaido Bunkyo University, Eniwa, Hokkaido Japan; 4 Department of Health Sciences, School of Medicine, Hokkaido University, Sapporo, Hokkaido Japan

**Keywords:** Neuroscience, Stem cells

## Abstract

Alzheimer’s disease (AD) is characterized by the accumulation of amyloid-β and tau. We previously reported that administration of bone marrow mesenchymal stem cells (BM-MSCs) ameliorates diabetes-induced cognitive impairment by transferring exosomes derived from these cells into astrocytes. Here, we show that intracerebroventricularly injected BM-MSCs improve cognitive impairment in AD model mice by ameliorating astrocytic inflammation as well as synaptogenesis. Although AD model mice showed an increase in NF-κB in the hippocampus, BM-MSC-treated AD model mice did not show this increase but showed an increase in levels of microRNA (miR)-146a in the hippocampus. Intracerebroventricularly injected BM-MSCs were attached to the choroid plexus in the lateral ventricle, and thus, BM-MSCs may secrete exosomes into the cerebrospinal fluid. In vitro experiments showed that exosomal miR-146a secreted from BM-MSCs was taken up into astrocytes, and an increased level of miR-146a and a decreased level of NF-κB were observed in astrocytes. Astrocytes are key cells for the formation of synapses, and thus, restoration of astrocytic function may have led to synaptogenesis and correction of cognitive impairment. The present study indicates that exosomal transfer of miR-146a is involved in the correction of cognitive impairment in AD model mice.

## Introduction

Hallmarks of Alzheimer’s disease (AD), the most common type of dementia, are amyloid-β (Aβ) plaques and tau tangles^1,2^. However, several trials of drugs targeting Aβ including β-secretase inhibitors failed to slow cognitive decline, even though Aβ plaque formation was reduced^3^. On the other hand, some post-mortem studies showed the existence of cognitively intact individuals with definite pathological features of AD^4^. In addition, we revealed that astrocytic function is associated with the maintenance of cognitive function and that a beneficial type of astrocytes protects neuronal activity from the toxicity of Aβ and tau^5^.

Stem cell therapy has emerged as a novel treatment for many diseases. Bone marrow (BM)- or adipose-derived mesenchymal stem cells (MSCs) have been proposed to reduce the level of Aβ by activating microglia^6,7^. Currently, clinical trials using autologous or allogeneic MSCs for AD are ongoing throughout the world^8^. However, how MSCs repair the damaged astrocytes in AD models has not been investigated in detail.

In a previous study, we reported that BM-MSC administration ameliorates diabetes-induced cognitive impairment^9^. Hyperglycemia induces damage to astrocytes by increasing oxidative stress^10^. BM-MSC-derived exosomes are taken up into astrocytes and can repair diabetes-induced astroglial damage by ameliorating mitochondrial abnormalities^9^. In addition, we previously showed that microRNA (miR)-146a, which is contained in exosomes that are secreted from BM-MSCs, ameliorates diabetes-induced astroglial inflammation via transfer into astroglia^11^. miR-146a is an anti-inflammatory microRNA that down-regulates NF-κB activity by repressing interleukin-1 receptor-associated kinase 1 (IRAK1) and tumor necrosis factor receptor-associated factor 6 (TRAF6)^11^.

BM-MSC-derived exosomes also contain other miRNAs, including miR-133b, which improves neuronal plasticity in a stroke model^12^ and miR-21, which promotes cell survival in an intracerebral hemorrhage model^13^. MSC-derived exosomes ameliorate neural impairment by suppressing inducible nitric oxide synthase expression in AD mice^14^. However, which miRNA contained in BM-MSC-derived exosomes modulates Aβ-associated pathology is unknown. Aβ also induces oxidative stress and NF-κB signaling in astrocytes^15^. Thus, here we investigated whether BM-MSCs can improve cognitive impairment in AD model mice and how they repair damaged astrocytes, with a focus on miR-146a. The results of the present study may suggest a novel therapeutic intervention for cognitive impairment in AD and the mechanism of action.

## Results

### BM-MSC administration improved learning and memory impairment in amyloid precursor protein/presenilin 1 (APP/PS1) mice

**Figure 1:**
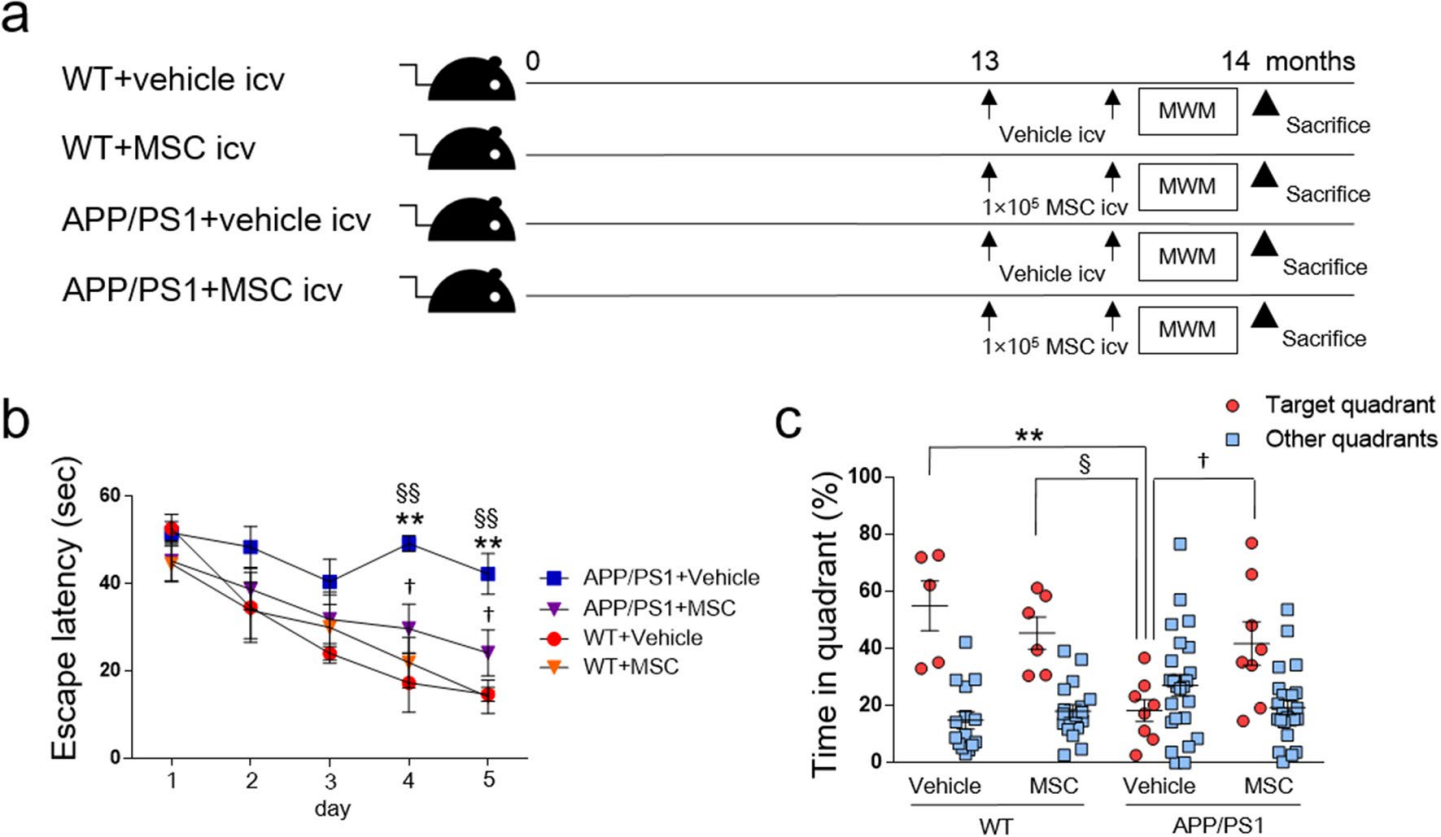
The effects of intracerebroventricular injection of BM-MSCs on AD model mice. (**a**) Experimental protocol. At 13 months old, WT and APP/PS1 mice were injected intracerebroventricularly with 1 × 10^5^ BM-MSCs/mouse or vehicle two times at a 2-week interval. (**b**) The hidden platform test of the MWM test. n = 5–8/group. Values are the means of four trials per day ± SEM. WT + vehicle, *F*(1.835, 7.341) = 12.10,* P* = 0.0052; WT + MSC, *F*(2.099, 10.50) = 7.895,* P* = 0.0076; APP/PS1 + vehicle, *F*(2.071, 14.49) = 1.829,* P* = 0.1951; APP/PS1 + MSC, *F*(2.834, 19.84) = 4.478,* P* = 0.0160. One-way ANOVA for repeated measures, by group. **P < 0.01, WT + vehicle vs. APP/PS1 + vehicle; ^§§^*P* < 0.01, WT + MSC vs. APP/PS1 + vehicle; ^†^*P* < 0.05, APP/PS1 + vehicle vs. APP/PS1 + MSC. Two-way ANOVA, Tukey post-hoc test, at each day. (**c**) The probe test of the MWM test. n = 5–8/group. Values are the means ± SEM. **P < 0.01, WT + vehicle vs. APP/PS1 + vehicle; ^§^*P* < 0.05, WT + MSC vs. APP/PS1 + vehicle; †*P* < 0.05, APP/PS1 + vehicle vs. APP/PS1 + MSC, two-way ANOVA, Tukey post-hoc test.

In APP/PS1 mice, learning and memory deficits were not observed at 10 months of age (Suppl. Fig. 1a), but were observed in 13-month-old mice (Suppl. Fig. 1b). Thus, we intravenously injected 1 × 10^4^ BM-MSCs/g body weight into 13-month-old APP/PS1 mice for treatment, four times at 2-week intervals, according to a previous study^9^ (Suppl. Fig. 1c). However, no cognitive improvement was observed (Suppl. Fig. 1d). We hypothesized that local injection may be more effective, and thus, intracerebroventricular administration of 1 × 10^5^ BM-MSCs/mouse was performed in 13-month-old APP/PS1 mice, two times at a 2-week interval (Fig. 1a). Four groups of mice were studied: wild-type (WT) mice given vehicle or MSCs (WT + vehicle, WT + MSC) and APP/PS1 mice given vehicle or MSCs (APP/PS1 + vehicle, APP/PS1 + MSC). At 1 week after the last injection, Morris water maze (MWM) tests were conducted, and mice were sacrificed at 14 months old. In the hidden platform test of the MWM test, WT + vehicle, WT + MSC, and APP/PS1 + MSC showed shorter escape latencies; however, the swimming time of the APP/PS1 + vehicle group was not shortened (Fig. 1b). At days 4 and 5, APP/PS1 + vehicle mice showed significantly prolonged escape latency compared to both WT + vehicle and WT + MSC mice, and this extended latency was shortened in APP/PS1 + MSC mice (Fig. 1b). We found no difference in the swimming speed among the four experimental groups (WT + vehicle; 0.1688 ± 0.0064 ms^−1^, WT + MSC; 0.1783 ± 0.0070 ms^−1^, APP/PS1 + vehicle; 0.1755 ± 0.0150 ms^−1^, APP/PS1 + MSC; 0.1738 ± 0.0143 ms^−1^). In the probe test, the percentage of time spent in the target quadrant was decreased in APP/PS1 + vehicle mice compared to WT + vehicle and WT + MSC mice, and this decrease was improved in APP/PS1 + MSC mice (Fig. 1c).

### BM-MSCs did not affect the area of Aβ or neuronal loss in AD model mice

**Figure 2:**
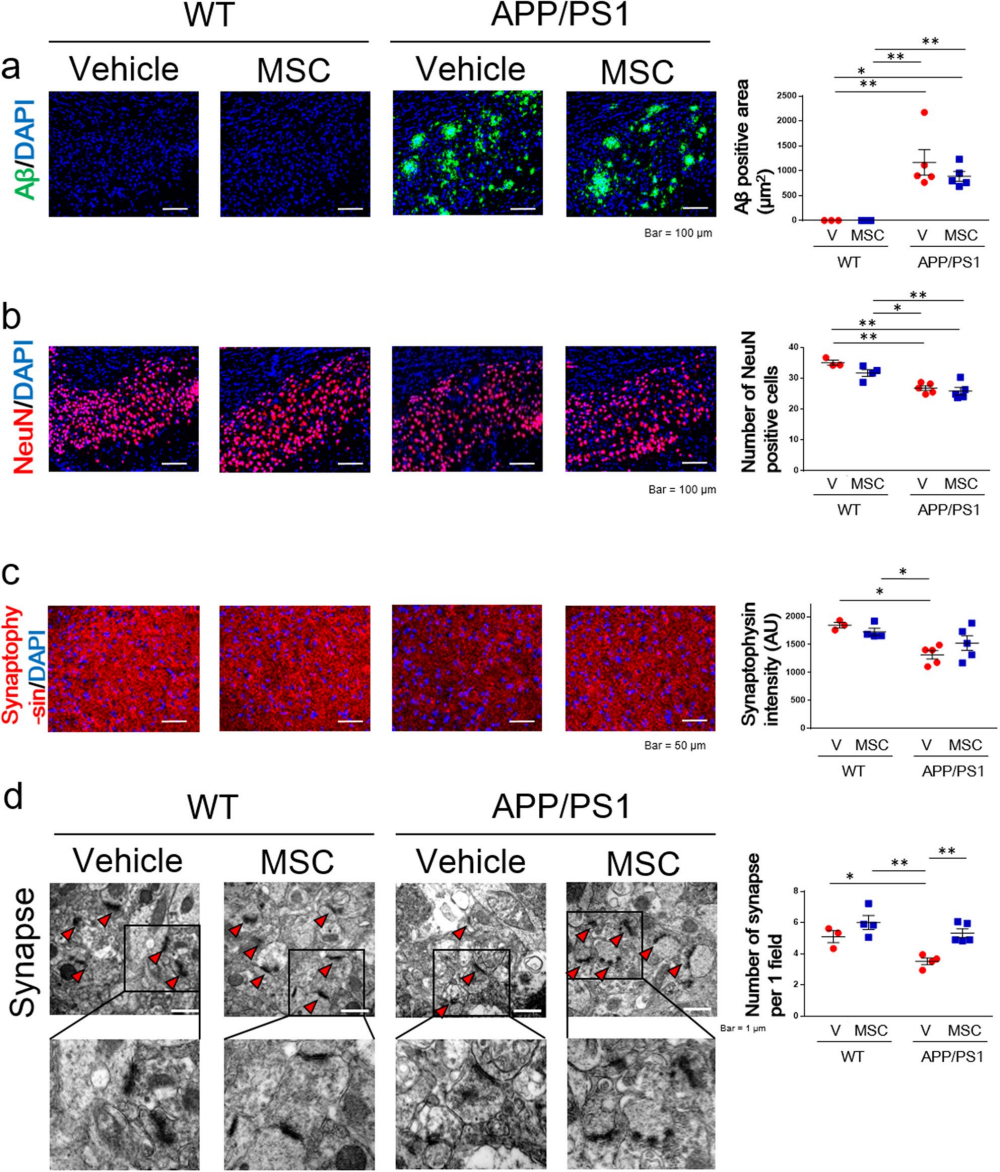
Immunohistochemical and electron microscopic analysis of the subiculum area in BM-MSC-treated mice. (**a**) The Aβ-positive area. n = 3–5/group. (**b**) The number of NeuN-positive cells. n = 3–5/group. (**c**) The intensity of synaptophysin. n = 3–5/group. (**d**) The number of synapses in the subiculum area. n = 3–5/group. Values are the means ± SEM. *P < 0.05, **P < 0.01, two-way ANOVA, Tukey post-hoc test.

We investigated the mechanisms by which BM-MSCs improve cognitive impairment in AD model mice. The area of Aβ in the subiculum region was compared in mice treated with BM-MSCs (Fig. 2a). The area of Aβ in the APP/PS1 + vehicle group was significantly increased compared to the WT + vehicle and WT + MSC groups, and this increase was not down-regulated in the APP/PS1 + MSC group (Fig. 2a).

The number of NeuN-positive neurons in the subiculum area was also evaluated in mice treated with BM-MSCs (Fig. 2b). The number of neurons in the APP/PS1 + vehicle group was significantly decreased compared to the WT + vehicle and WT + MSC groups, and this decrease was not improved in the APP/PS1 + MSC group (Fig. 2b).

### Effects of BM-MSCs on synaptic density in AD model mice

We examined synaptic density in the subiculum area by staining sections with the synaptic marker, synaptophysin. The intensity of synaptophysin staining in the APP/PS1 + vehicle group was significantly down-regulated compared to the WT + vehicle and WT + MSC groups, and no significant decrease was observed in the APP/PS1 + MSC group compared to the WT + vehicle and WT + MSC groups (Fig. 2c). The density of synapses in the subiculum area was also assessed with electron microscopy by counting the number of synapses directly. The APP/PS1 + vehicle group showed a decreased number of synapses compared to the WT + vehicle and WT + MSC groups, and this decrease was improved in the APP/PS1 + MSC group (Fig. 2d).

### BM-MSCs decreased glial fibrillary acidic protein (GFAP)- and tumor necrosis factor (TNF)α-positive areas in astrocytes in AD model mice

**Figure 3:**
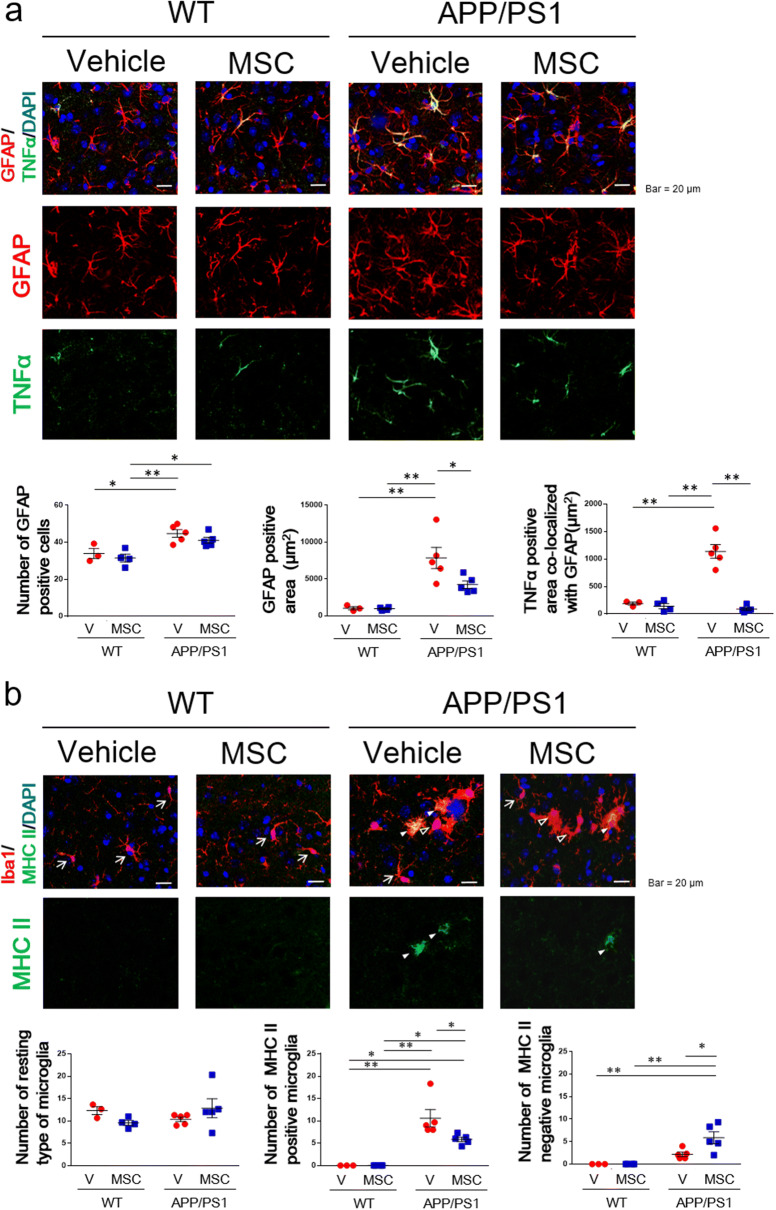
Immunohistochemical analysis of glial cells in the subiculum area in BM-MSC-treated mice. (**a**) The number of GFAP-positive cells, the GFAP-positive area, and the TNFα-positive area. n = 3–5/group. (**b**) The number of the resting type of microglia (arrows), the number of the M1 type of microglia (white arrowheads), and the number of the activated type of microglia, which are negative for MHC class II (black arrowheads). n = 3–5/group. *P < 0.05, **P < 0.01, two-way ANOVA, Tukey post-hoc test.

Co-staining for GFAP and TNFα was performed to evaluate astrocytic characteristics in the subiculum region in mice treated with BM-MSCs (Fig. 3a). The number of GFAP-positive cells in the APP/PS1 + vehicle group was significantly increased compared to the WT + vehicle and WT + MSC groups, and this increase was not down-regulated in the APP/PS1 + MSC group (Fig. 3a). The GFAP-positive area in the APP/PS1 + vehicle group was significantly increased compared to the WT + vehicle and WT + MSC groups, and this increase was decreased in the APP/PS1 + MSC group (Fig. 3a). Moreover, the positive area of TNFα that co-localized with GFAP was significantly higher in the APP/PS1 + vehicle group than the WT + vehicle and WT + MSC groups, and this increase was down-regulated in the APP/PS1 + MSC group (Fig. 3a).

### BM-MSCs decreased the M1 type of activated microglia and increased the M2 type of activated microglia in AD model mice

Iba1 positive cells were divided into resting microglia, with a small cell body (area < 70 µm^2^) and long branching processes, and activated microglia, with a large cell body (area > 70 µm^2^) and less ramification of branches. In WT mice, Iba1 positive cells include only resting microglia (arrows in Fig. 3b), while in APP/PS1 mice, Iba1 positive cells include both resting (arrows in Fig. 3b) and activated microglia (arrow heads in Fig. 3b). We found no difference in the number of resting microglia among the four groups (Fig. 3b). Then we performed co-staining of Iba1 and major histocompatibility complex (MHC) class II to evaluate the characteristics of activated microglia (Fig. 3b). The number of MHC class II-positive/Iba1 positive cells, which are the M1 type of activated microglia, was significantly higher in the APP/PS1 + vehicle group than the WT + vehicle and WT + MSC groups, and this increase was ameliorated in the APP/PS1 + MSC group (Fig. 3b). On the other hand, the number of MHC class II-negative/Iba1positive cells, which are suggested to be the M2 type of activated microglia, was significantly higher in the APP/PS1 + MSC group than the WT + vehicle, WT + MSC, and APP/PS1 + vehicle groups (Fig. 3b).

### Paul Karl Horan (PKH)-labeled BM-MSCs were attached to the choroid plexus (CP) after intracerebroventricular injection in APP/PS1 mice

**Figure 4:**
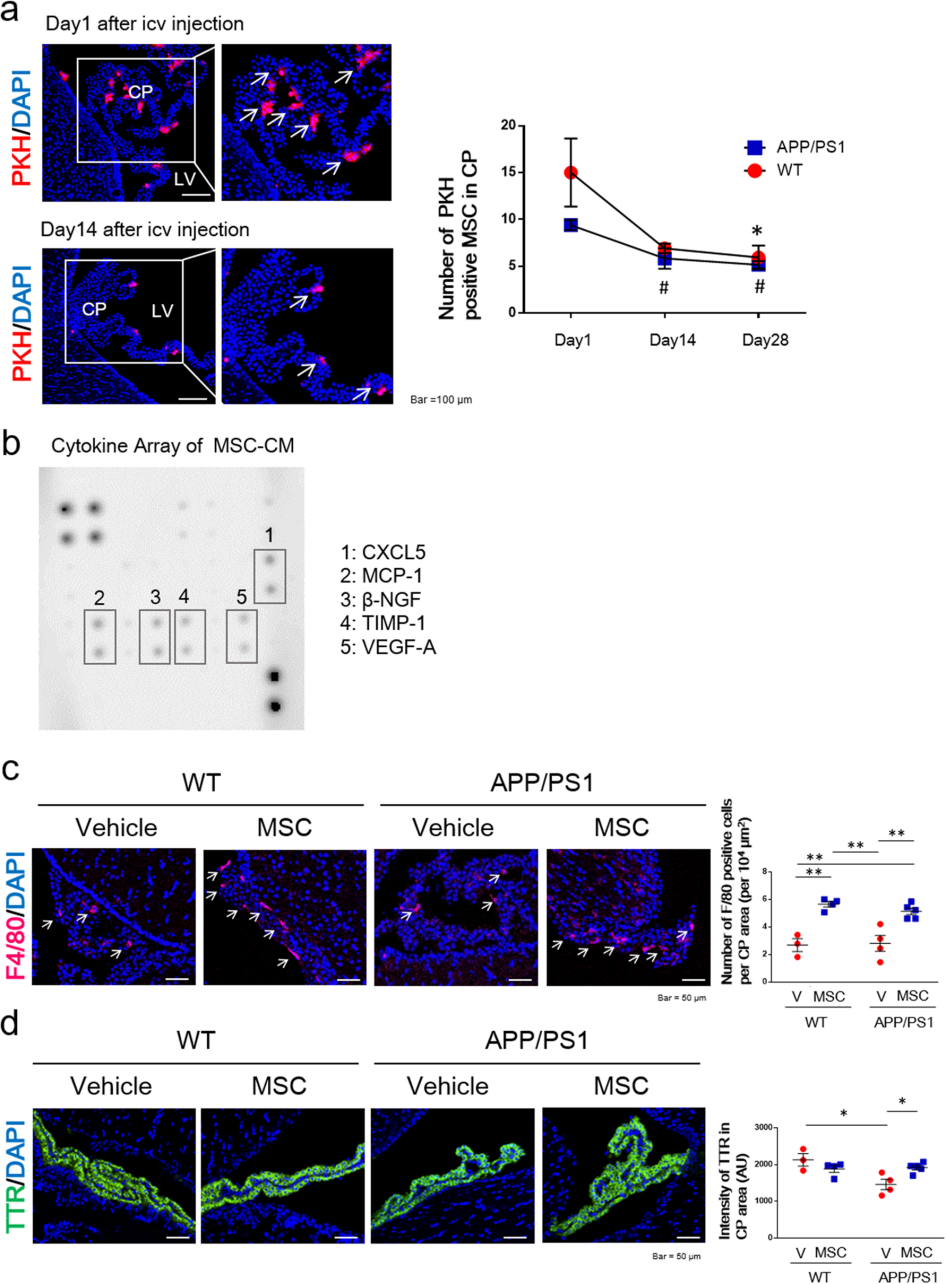
The distribution of BM-MSCs after intracerebroventricular injection. (**a**) Images of PKH-labeled BM-MSCs attached to the choroid plexus (CP) in the lateral ventricle (LV) after intracerebroventricular injection at days 1 and 14 (left panel). The number of PKH-labeled BM-MSCs (arrows) attached to the CP after intracerebroventricular injection at days 1, 14, and 28 (right panel). n = 3/group. Values are the means ± SEM. *P < 0.05, WT day 1 vs. WT day 28; #*P* < 0.05, APP/PS1 day 1 vs. day 14, day 1 vs. day 28, one-way ANOVA, Tukey post-hoc test. (**b**) The results of the rat cytokine array with CM from BM-MSCs. (**c**) The number of F4/80-positive cells in BM-MSC-treated mice. Arrows point to F4/80-positive cells. n = 3–5/group. Values are the means ± SEM. **P < 0.01, two-way ANOVA, Tukey post-hoc test. (**d**) The intensity of TTR in BM-MSC-treated mice. n = 3–5/group. Values are the means ± SEM. *P < 0.05, two-way ANOVA, Tukey post-hoc test.

To examine the distribution of BM-MSCs, 1 × 10^5^ PKH-labeled BM-MSCs were injected into the lateral ventricle of both WT and APP/PS1 mice. PKH binds to the cell membrane and is used for long-term cell tracking^16^. PKH-labeled BM-MSCs attached to the CP were observed after the injection in WT and APP/PS1 mice (Fig. 4a). Compared to day 1, the number of PKH-labeled BM-MSCs was significantly decreased at day 28 in the WT group, and these cells were significantly decreased at days 14 and 28 in the APP/PS1 group. We found no difference in the number of PKH-labeled BM-MSCs between the WT and APP/PS1 groups at days 1, 14, or 28 (Fig. 4a).

### The cytokine array showed that BM-MSCs secrete various proteins

To examine humoral factors released from BM-MSCs, we performed a cytokine array of conditioned medium (CM) from BM-MSCs grown in culture for 24 h (Fig. 4b). The cytokine array showed that C-X-C Motif Chemokine Ligand 5 (CXCL5), monocyte chemoattractant protein-1 (MCP-1), beta-nerve growth factor (β-NGF), tissue inhibitor of metalloproteinase-1 (TIMP-1), and vascular endothelial growth factor-A (VEGF-A) were mainly detected in MSC-CM.

### The number of F4/80-positive macrophages and the intensity of transthyretin (TTR) were increased in the CP by BM-MSC injection into APP/PS1 mice

Because CXCL5 and MCP-1 are chemoattractant proteins that induce the migration of monocytes and neutrophils, we evaluated the number of F4/80-positive macrophages that were attracted to the CP area. In both WT and APP/PS1 mice, the number of F4/80-positive cells was increased in the CP area in MSC-treated mice compared to vehicle-treated mice (Fig. 4c).

Because the expression of TTR is decreased in the CP area in AD mice^17^, we evaluated the intensity of TTR in MSC-treated mice. The intensity of TTR in the APP/PS1 + vehicle group was significantly decreased compared to the WT + vehicle groups, and this decline was increased in the APP/PS1 + MSC group (Fig. 4d).

### The expression of miR-146a, IRAK1, TRAF6, and NF-κB in the hippocampus of AD model mice treated with BM-MSCs

**Figure 5:**
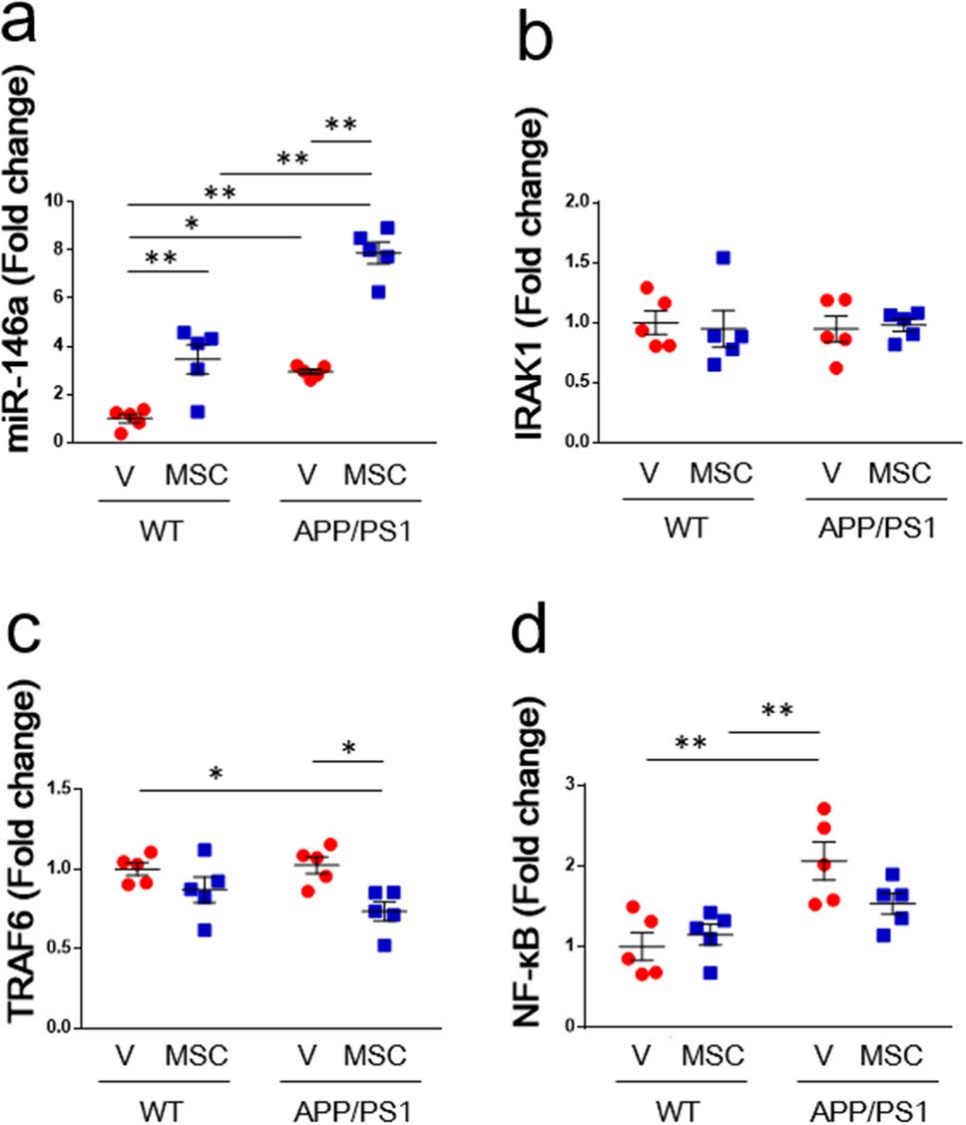
Analysis of miRNA and mRNA in the hippocampus. The expression of (**a**) miR-146a, (**b**) IRAK1, (**c**) TRAF6, and (**d**) NF-κB in vehicle- or BM-MSC-treated mice. n = 5/group. Values are the means ± SEM. *P < 0.05, **P < 0.01, two-way ANOVA, Tukey post-hoc test.

We previously showed that exosomes secreted from BM-MSCs ameliorates astrocytic inflammation in a diabetes model^9^. BM-MSC derived exosomes suppress the expression of NF-κB by transferring its containing miR-146a into astrocytes^11^. Thus, we hypothesized that BM-MSCs attached to the CP may secret exosomes and ameliorate astrocytic inflammation in AD model by transferring exosomal miR-146a into hippocampus. We analyzed the expression of miR-146a in the hippocampus and the mRNA expression of IRAK1, TRAF6 and NF-κB in the subiculum in AD model mice treated with BM-MSCs. The expression of miR-146a in the hippocampus was significantly up-regulated in the APP/PS1 + vehicle group compared to the WT + vehicle group (Fig. 5a). Moreover, the expression of miR-146a in the hippocampus was significantly up-regulated in the APP/PS1 + MSC group compared to the APP/PS1 + vehicle group (Fig. 5a). We found no difference in the expression of IRAK1, a molecular target of miR-146a, in the subiculum among the four groups (Fig. 5b). However, the expression of TRAF6 in the subiculum area was significantly decreased in the APP/PS1 + MSC group compared to the WT + vehicle and APP/PS1 + vehicle groups (Fig. 5c). The expression of NF-κB in the subiculum area was significantly increased in the APP/PS1 + vehicle group compared to the WT + vehicle and WT + MSC groups. However, no significant increase in NF-κB was observed in the APP/PS1 + MSC group compared to the WT + vehicle and WT + MSC groups (Fig. 5d).

### BM-MSC-derived exosomal miR-146a was taken up into astrocytes and exerted anti-inflammatory effects

**Figure 6:**
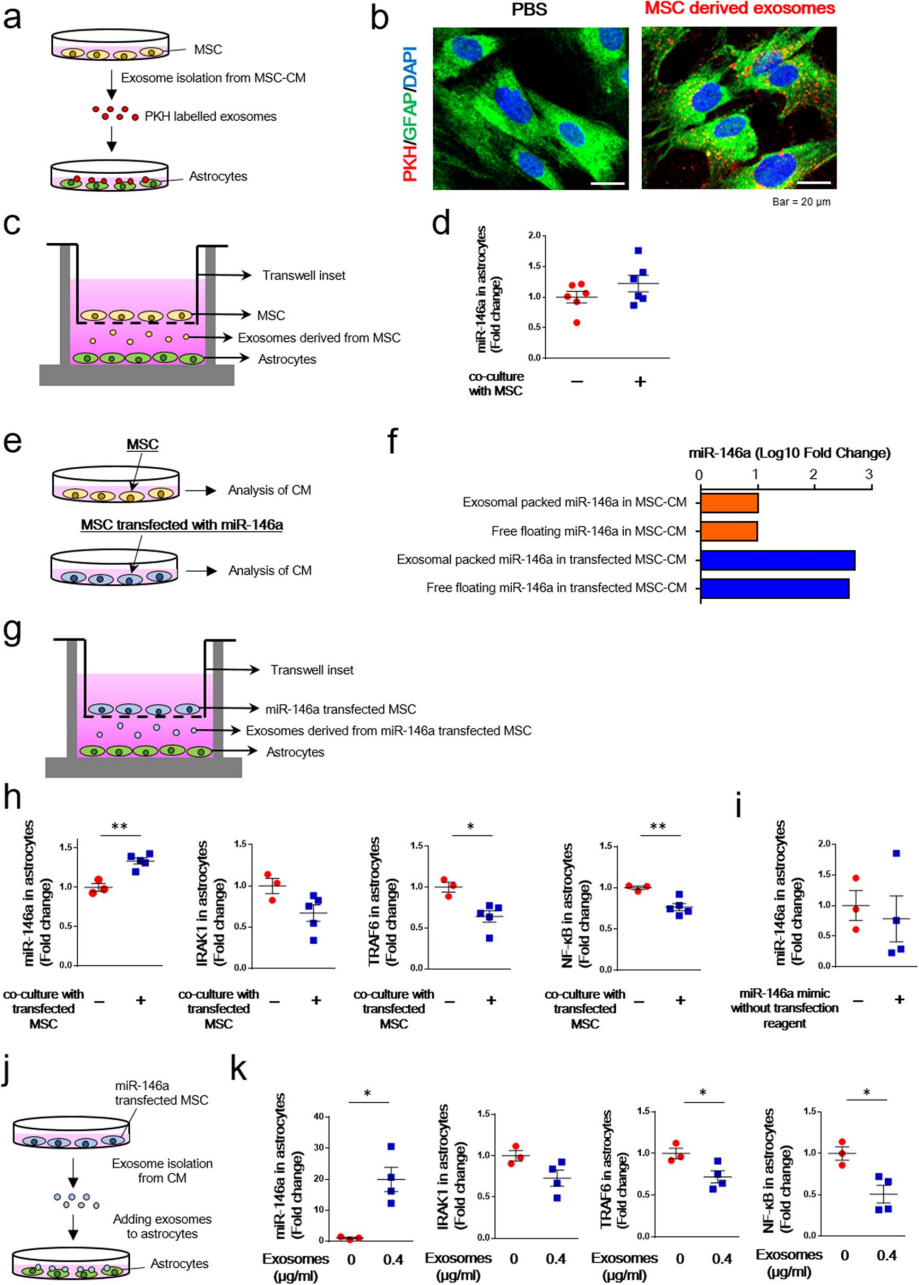
Analysis of the effect of BM-MSC-derived exosomes. (**a**) Schematic representation of the exosome uptake experiment. BM-MSC-derived exosomes were labeled with PKH and incubated with astrocytes. (**b**) BM-MSC-derived exosomes were internalized by GFAP-positive astrocytes. PBS was used as a control. (**c**) A transwell co-culture assay with BM-MSCs (top well) and astrocytes (bottom well). To inhibit cell–cell contact, a 0.4-µm porous membrane was used as an insert. (**d**) The expression of miR-146a in astrocytes with or without BM-MSCs. n = 6/group. Values are the means ± SEM. Two-tailed unpaired *t*-test. (**e**) Schematic representation of the analysis of BM-MSC-derived CM and miR-146a-transfected BM-MSC-derived CM. (**f**) The levels of exosomes packed with miR-146a as well as free-floating miR-146a were evaluated in both non-transfected BM-MSC-derived CM and miR-146a-transfected BM-MSC-derived CM. n = 1/group. (**g**) A transwell co-culture assay with miR-146a-transfected BM-MSCs (top well) and astrocytes (bottom well). To inhibit cell–cell contact, a 0.4-µm porous membrane was used as an insert. (**h**) The expression of miR-146a, IRAK1, TRAF6, and NF-κB in astrocytes with or without transfected BM-MSCs. n = 3–5/group. Values are the means ± SEM. *P < 0.05, **P < 0.01, two-tailed unpaired *t*-test. (**i**) The expression of miR-146a in astrocytes after adding the miR-146a mimic without transfection reagent. n = 3–4/group. Values are the means ± SEM. Two-tailed unpaired *t*-test. (**j**) Exosomes isolated from the CM of miR-146a-transfected BM-MSCs were added to astrocytes. (**k**) The expression of miR-146a, IRAK1, TRAF6, and NF-κB in astrocytes with or without exosomes that were isolated from the CM of miR-146a-transfected BM-MSCs. n = 3–4/group. Values are the means ± SEM. *P < 0.05, two-tailed unpaired *t*-test.

To examine whether BM-MSC-derived exosomes were taken up into astrocytes, we performed in vitro experiments. First, we isolated exosomes from the CM of cultured BM-MSCs, labeled them with PKH, and added these labeled exosomes to cultured astrocytes (Fig. 6a). PKH-labeled exosomes were internalized by GFAP-positive astrocytes (Fig. 6b).

Next, we examined whether miR-146a contained in BM-MSC-derived exosomes ameliorated astrocytic inflammation. BM-MSCs were co-cultured with astrocytes using a transwell culture plate, and expression of miR-146a in astrocytes was measured after co-culture with and without BM-MSCs (Fig. 6c). We found no difference in miR-146a expression in astrocytes between these two groups (Fig. 6d). In the next experiment, we transfected BM-MSCs with miR-146a and measured the levels of miR-146a packed within exosomes and free-floating miR-146a in the CM from transfected BM-MSCs and non-transfected BM-MSCs (Fig. 6e,f). In the CM of miR-146a-transfected BM-MSCs, the levels of miR-146a packed in exosomes and free-floating miR-146a were higher than those in the CM of non-transfected BM-MSCs (Fig. 6f).

Then, we performed co-culture of miR-146a-transfected BM-MSCs with astrocytes using a transwell culture plate, and the expression of miR-146a in astrocytes was measured (Fig. 6g). The miR-146a levels in astrocytes were significantly increased after co-culture with transfected BM-MSCs compared to those without transfected BM-MSCs (Fig. 6h). In addition, the expression of TRAF6 and NF-κB in astrocytes was decreased after co-culture with transfected BM-MSCs compared to those without transfected BM-MSCs (Fig. 6h). To rule out the effect of free-floating miR-146a derived from BM-MSCs on the expression of miR-146a in astrocytes, we applied a miR-146a mimic to the CM of cultured astrocytes and measured the expression of miR-146a in astrocytes. We found no difference in miR-146a expression in astrocytes cultured with and without the miR-146a mimic (Fig. 6i).

To confirm the involvement of miR-146a packed in exosomes derived from BM-MSCs in ameliorating astrocytic inflammation, we isolated exosomes from the CM of cultured BM-MSCs transfected with miR-146a and applied the CM to cultured astrocytes (Fig. 6j). When exosomes (0.4 µg/ml) were added, the expression of miR-146a in astrocytes was significantly increased compared to that in astrocytes without addition of exosomes (Fig. 6k). In addition, the expression of TRAF6 as well as NF-κB was significantly decreased in astrocytes with added exosomes (0.4 µg/ml) compared to those without added exosomes (Fig. 6k).

## Discussion

To the best of our knowledge, this is the first report to reveal that BM-MSCs improve cognitive impairment in an AD model by transferring exosomal miR-146a into astrocytes. Although the treatment with BM-MSCs did not change the Aβ-positive area or neuronal number in the subiculum area, improvement in other pathological conditions such as an increase in synaptic density, amelioration of inflammation in astrocytes, and a decrease in the ratio of M1/M2 activated microglia was observed in AD model mice treated with BM-MSCs. Intracerebroventricularly injected BM-MSCs attached to the CP. Thus, BM-MSCs may secrete exosomes into the cerebrospinal fluid (CSF). PCR analysis of mRNA levels in the hippocampus showed that vehicle-treated AD model mice had increased levels of NF-κB, whereas BM-MSC-treated AD model mice did not show an increase in NF-κB. In addition, up-regulated expression of miR-146a as well as down-regulated expression of TRAF6 were observed in AD model mice treated with BM-MSCs. When BM-MSC-derived exosomes labeled with PKH were added to cultured astrocytes, internalization of exosomes by astrocytes was observed. In addition, when co-culture of miR-146a-transfected BM-MSCs with astrocytes was performed in a transwell culture plate, up-regulated expression of miR-146a as well as down-regulated expression of TRAF6 and NF-κB were observed in astrocytes. Moreover, when exosomes collected from the CM of miR-146a-transfected BM-MSCs were added to the CM of astrocytes, up-regulated expression of miR-146a as well as down-regulated expression of TRAF6 and NF-κB were observed in astrocytes. Thus, BM-MSC treatment corrected the cognitive impairment in AD mice by transferring exosomal miR-146a to suppress the inflammation of astrocytes, resulting in promotion of synaptogenesis.

Because cognitive impairment was detected in 13-month-old APP/PS1 mice, BM-MSC treatment was started at this time point. According to a previous study^9^, we first intravenously injected 1 × 10^4^ BM-MSCs/g body weight into 13-month-old APP/PS1 mice; however, cognitive function was not improved. AD model mice exhibit more severe cognitive impairment and exacerbated cerebrovascular inflammation than diabetic model mice^18^. Therefore, intravenous injection appeared to not be sufficient to improve cognitive impairment in APP/PS1 mice.

Following intracerebroventricular injection of BM-MSCs, learning and memory abnormalities were improved compared to vehicle-treated APP/PS1 mice. In a previous study, injection of Aβ_25-35_ oligomers was used to create an AD model in which cognitive impairment was prevented by intracerebroventricular injection of BM-MSCs^19^. However, AD pathology is more complicated than merely Aβ deposition^20^. We used APP/PS1 mice as an AD model and investigated the effects of BM-MSCs on not only cognitive function but also on pathological changes in the brain.

APP/PS1 mice showed increased accumulation of Aβ in the subiculum of the hippocampus. However, BM-MSCs did not reduce the positive area of Aβ. Previously, we found that cognitive function is associated with synaptic density, rather than the accumulation of Aβ and tau in a human post-mortem study^5^. Thus, factors other than Aβ accumulation may have contributed to the improvement in cognitive function in mice treated with BM-MSCs.

The reduced number of NeuN-positive neurons in APP/PS1 mice was not reversed by BM-MSC treatment. In contrast, the decreased synaptophysin intensity that was observed in APP/PS1 mice was not found in APP/PS1 mice treated with BM-MSCs. In addition, the number of synapses was increased in BM-MSC-treated mice at the ultrastructural level. These findings suggest that evaluation of synaptophysin intensity is less sensitive compared to direct counting of the synaptic number seen with electron microscopy. Because cognitive impairment is associated with reduced synaptic density, the improvement in cognitive function in mice treated with BM-MSCs may be related to the restoration of synaptic density.

The effects of BM-MSCs on astrocytes were evaluated with GFAP staining. The number of astrocytes in the APP/PS1 mice treated with vehicle was significantly increased compared to WT groups; however, this increase was not ameliorated in APP/PS1 mice treated with BM-MSCs. In contrast, the increase in the GFAP-positive area in APP/PS1 mice was ameliorated by BM-MSC treatment. Because reactive astrocytes with increased expression of GFAP release inflammatory cytokines^21^ and impair synaptic plasticity^22^, the reduction in the GFAP-positive area may have been associated with improvement in astrocytic function.

Moreover, the increase in the TNFα-positive area that co-localized with GFAP in APP/PS1 mice was down-regulated by BM-MSC treatment. BM-MSCs reduce astrocyte-derived cytokines such as TNFα and inducible nitric oxide synthase^23^. Because the reduction in TNFα expression in astrocytes was prominent in APP/PS1 mice treated with BM-MSCs, recovery of astrocytic function may be a key factor for synaptogenesis and improvement in cognitive function.

The effects of BM-MSCs on microglia were evaluated with Iba1 staining. All of Iba1 positive microglia was resting type in WT mice, while activating type of microglia was increased in APP/PS1 mice. This phenomenon is consistent with previous study that showed resting type of microglia were converted into activated type in response to neuroinflammatory stimuli including Aβ^24^. Activated microglia are divided into two types, one is pro-inflammatory type (M1) which is MHC II-positive and the other is alternative protective type (M2), which is MHC II-negative. In APP/PS1 mice the number of MHC II-positive M1 type of microglia was significantly increased in APP/PS1 mice compared to WT groups, and this increase was down-regulated by BM-MSC treatment. On the other hand, BM-MSC treatment up-regulated the number of MHC II-negative M2 type of microglia in APP/PS1 mice. The M2 phenotype facilitates Aβ clearance by phagocytosis and secretion of anti-inflammatory cytokines, such as transforming growth factor-β and interleukin-10^25,26^. Although Aβ accumulation was not changed by BM-MSC treatment, the increased number of cells with the M2 phenotype may have contributed to tissue repair via an anti-inflammatory effect.

When PKH-labeled BM-MSCs were injected intracerebroventricularly, BM-MSCs attached to the CP in the lateral ventricle. This finding suggests that MSCs secrete humoral factors including exosomes into the CSF. To examine humoral factors released from BM-MSCs, we performed a cytokine array of CM derived from BM-MSCs. The cytokine array showed that CXCL5, MCP-1, β-NGF, TIMP-1, and VEGF-A were mainly detected in MSC-CM. The roles of β-NGF, TIMP-1, and VEGF-A include sprouting of axons from degenerating neurons^27^, inhibition of the activities of matrix metalloproteinases^28^, and maintenance of blood vessels^29^, respectively. Because AD brain shows axonal degeneration^30^, increased matrix metalloproteinase-9 expression that facilitates tau aggregation^28^, and impaired cerebral blood vessel structure^31^, β-NGF, TIMP-1, and VEGF-A may be involved in regulation of these pathological conditions.

CXCL5 and MCP-1 are chemoattractant proteins that induce the migration of monocytes and neutrophils^32,33^. We observed that the number of F4/80-positive macrophages was increased in the CP area in MSC-treated mice. Because the number of PKH-labeled BM-MSCs was decreased at 14 and 28 days after injection in APP/PS1 mice, these macrophages may contribute to phagocytosis of MSCs^34^. Moreover, we found that a decline in TTR expression was corrected by BM-MSC treatment. The expression of TTR plays a role in maintaining the blood–CSF barrier and is decreased in the CP area in AD mice^17,35^. Therefore, recovery of TTR expression in the CP by BM-MSC treatment may contribute to normalization of CP function and mitigation of AD pathology.

Although BM-MSCs release several proteins, we focused on exosomal miRNA secreted from BM-MSCs, because miRNAs regulate every aspect of cellular activity by regulating the expression of approximately 30–70% of genes^36^. Previously, we reported that exosomal miR-146a derived from BM-MSCs ameliorates astrocytic inflammation in a diabetes model^11^. Thus, we hypothesized that BM-MSCs may ameliorate the damage to astrocytes in an AD model by transferring exosomal miR-146a.

PCR analysis showed that the expression of miR-146a in the hippocampus in APP/PS1 mice was increased compared to WT + vehicle mice, and this increase was further up-regulated in APP/PS1 mice treated with BM-MSCs. miR-146a is a negative feedback regulator of NF-κB activation, and is up-regulated in the temporal cortex of AD patients^37^. Because APP/PS1 mice treated with vehicle showed an increase in miR-146a as well as an increase in NF-κB, the negative feedback regulation of miR-146a may not work well. miR-146a is localized in astrocytes^38^ and microglia^39^, and the M1 type of microglia shows higher expression of miR-146a compared to the M2 type^40^. In the present study, the number of M1 type microglia was decreased by BM-MSC treatment. This result suggests that the increase in miR-146a in the hippocampus may have been due to astrocytes. APP/PS1 mice treated with BM-MSCs showed an increase in miR-146a as well as a decrease in TRAF6 compared to APP/PS1 mice not treated with BM-MSCs. In addition, APP/PS1 mice treated with BM-MSCs did not show an increase in NF-κB compared to WT mice. This phenomenon indicates that further up-regulation of miR-146a in the hippocampus is needed for regulation of NF-κB expression in astrocytes.

We also examined the expression of miR-133b as well as RhoA in the hippocampus in BM-MSC treated mice. However, we found no difference among the four groups (Suppl. Fig. 3). Although exosomal miR-133b from BM-MSCs promotes neurological recovery from stroke by down-regulating the expression of RhoA^12^, these molecules seemed to not be involved in this study.

To examine the hypothesis that BM-MSCs ameliorate the damage to astrocytes by transferring exosomal miR-146a, we performed in vitro experiments. When PKH-labeled exosomes derived from BM-MSCs were added to the culture medium of astrocytes, PKH was internalized into GFAP-positive astrocytes. However in the experiments of co-culture of astrocytes and BM-MSCs, no increase was observed in miR-146a expression in astrocytes. This phenomenon may have been due to the short duration of the co-culture period (3 days) compared to in vivo conditions, in which PKH-labeled BM-MSCs were present for 28 days at the CP after injection. To resolve this problem, we transfected miR-146a into BM-MSCs and measured the expression of miR-146a packed in exosomes derived from these transfected BM-MSCs. Because higher levels of miR-146a were detected in exosomes derived from transfected BM-MSCs compared to non-transfected BM-MSCs, we used these cells for further experiments.

In the experiment of co-culture of astrocytes and miR-146a-transfected BM-MSCs, we observed up-regulation of miR-146a as well as down-regulation of TRAF6 and NF-κB in astrocytes. As a control experiment, we performed co-culture of astrocytes with miR-146a transfected BM-MSCs and those with non-transfected BM-MSCs. The effects on up-regulation of miR-146a expression and down-regulation of IRAK1 and NF-κB in astrocytes were observed in the former group than the latter group (Suppl. Fig. 4a, b).

Because the CM of cultured BM-MSCs contains free-floating miR-146a, we performed the following experiments to rule out the effects of free-floating miR-146a on astrocytes. We applied a miR-146a mimic to cultured astrocytes and measured the expression of miR-146a in astrocytes. On the other hand, we isolated exosomes from miR-146a-transfected BM-MSCs and applied them to cultured astrocytes. The results showed that the miR-146a mimic did not exert any effects on astrocytes, but isolated exosomes exerted positive effects on astrocytes. These findings indicate that miR-146a packed in exosomes but not free-floating miR-146a derived from BM-MSCs plays roles in ameliorating astrocytic inflammation.

**Figure 7:**
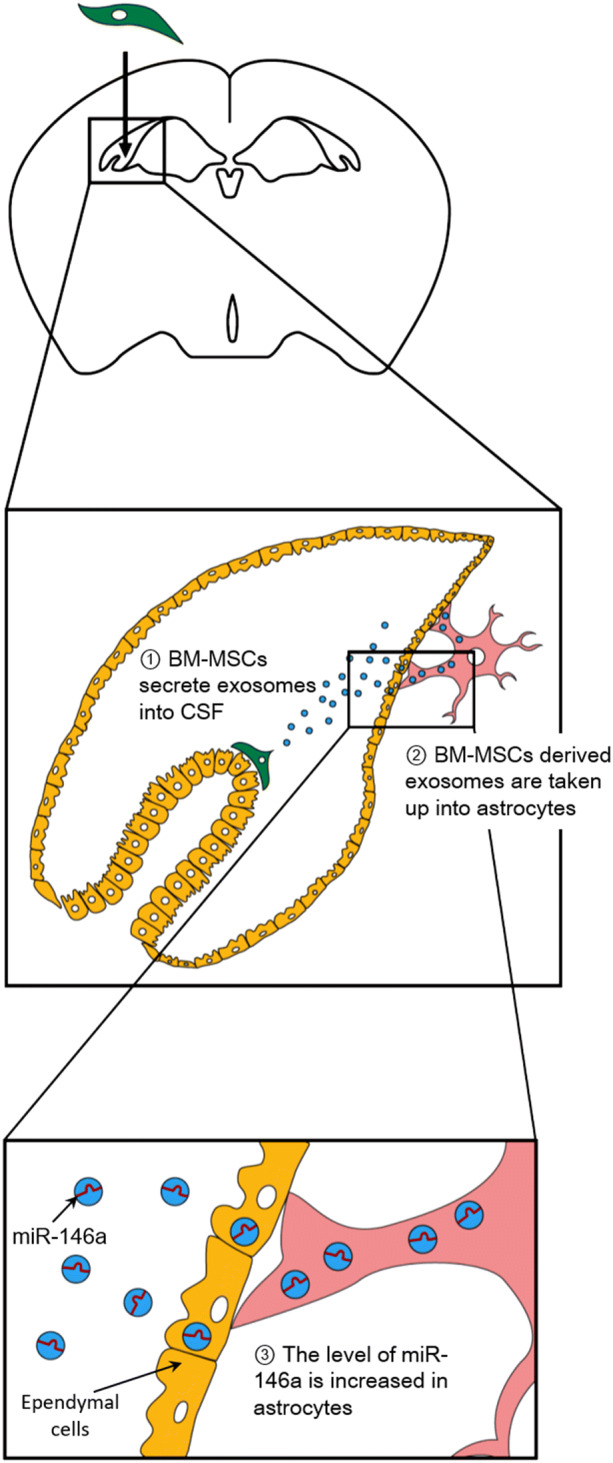
Schema of BM-MSC therapy. Intracerebroventricularly injected BM-MSCs attach to the CP and secrete exosomes containing miR-146a into the CSF. After astrocytes take up BM-MSC-derived exosomes, the level of miR-146a localized in astrocytes is increased. The increased miR-146a acts to suppress the inflammation of astrocytes in AD.

The present study shows that BM-MSCs improved cognitive impairment in AD model mice by transferring exosomal miR-146a into astrocytes. BM-MSCs increased the level of miR-146a in the hippocampus and attenuated astrocytic inflammation (Fig. 7). BM-MSCs may be a powerful therapeutic agent for treating cognitive impairment in AD. Although no effective treatment for AD has been developed so far, BM-MSCs may provide hope in the fight against not only AD but also other neurodegenerative diseases such as Parkinson disease^41^ and Down syndrome^42^ in which miR-146a in the brain is dysregulated.

## Methods

### Animals

All methods for animal experiments were performed in accordance with the approved guidelines of the Animal Experiment Committee of Sapporo Medical University (Sapporo, Japan). All experimental protocols were approved by the Animal Experiment Committee of Sapporo Medical University (approval #16–050, #17–065).

Male APP/PS1 mice were purchased from The Jackson Laboratory (Bar Harbor, ME, USA). APP/PS1 mice express a chimeric mouse/human APP (Mo/HuAPP695swe) and a mutant human PS1 (PS1-dE9)^43^. Male APP/PS1 mice were crossed with WT B6C3F1 females (Sankyo Lab Service Corp., Tokyo, Japan) to obtain the needed number of mice for experiments. Animals were maintained at temperature ranges of 21 °C to 24 °C and humidity ranges of 50% to 70%. Animals were housed under a 12-h light/12-h dark cycle with ad libitum access to food and water.

Every effort was made to minimize pain and distress. The animals were anesthetized with isoflurane inhalation during invasive procedures. The depth of anesthesia was checked by assessing toe pinch reflexes. At the end of the experiments, animals were euthanized with an overdose of pentobarbital (120 mg/kg intraperitoneal injection) or inhalation of excess isoflurane. After confirming the animal’s death by both the absence of breathing and cardiac arrest, all blood samples were obtained from the heart of animals.

### Isolation of BM-MSCs

Isolation of rat BM-MSCs was performed as described previously^9^. In brief, after male Sprague Dawley rats (Sankyo Lab Service Corp.) were sacrificed, BM was flushed out from removed tibias and femurs. Marrow was cultured in alpha-minimal essential medium (Thermo Fisher Scientific, Lafayette, CO, USA) with 15% fetal bovine serum (FBS) (CCB, NICHIREI BIOSCIENCE, Tokyo, Japan) and 1% penicillin streptomycin (PS) (Thermo Fisher Scientific). The medium was first changed 24 h after seeding and then twice weekly. BM-MSCs that were harvested after up to three passages and that were positive for CD90 and negative for CD45 and CD11b^44^ were used for further experiments.

### Intracerebroventricular injection of BM-MSCs

At 12 months of age, female APP/PS1 and WT mice were implanted with a stainless steel cannula (Eicom, Kyoto, Japan), as described previously^9^. The cannula (0.4 mm diameter) was placed at 0.4 mm posterior from the bregma, 1 mm right of the midline, and 2.5 mm deep to reach the right ventricle of the brain. At 13 months of age, female APP/PS1 and WT mice were injected with BM-MSCs (1 × 10^5^ BM-MSCs per mouse in 5 µl PBS (Thermo Fisher Scientific)) or vehicle (5 µl PBS) through the cannula with a Hamilton syringe (0.17 diameter) at a rate of 1 μl/min. After intracerebroventricular injection two times at a 2-week interval, the MWM test was conducted (Fig. 1a).

To evaluate the distribution of BM-MSCs, we injected WT and APP/PS1 mice at 13–14 months old with PKH-labeled BM-MSCs into the right ventricle via the cannula. BM-MSCs were stained with PKH26 (Sigma-Aldrich, St. Louis, MO, USA), according to the manufacturer’s protocol. Mice were sacrificed at 1, 14, and 28 days after injection of PKH-labeled BM-MSCs (1 × 10^5^ BM-MSCs per mouse in 5 µl PBS).

### MWM test

The MWM test was used to evaluate learning and memory, as described previously^9^. The mice were placed in a circular pool (1.2 m diameter) filled with water (25 ± 1 °C), and visual cues were placed around the pool. The MWM test includes a visible test (day 0), hidden platform test (days 1–5), and probe test (day 6). During the visible and hidden platform tests, the mice were released into the pool four times per day, and the escape latency (time to find the hidden platform) and the swimming speed were recorded. If the mice failed to find the platform in 60 s, they were picked up and placed on the platform for 15 s. During the probe test, they were released and allowed to swim freely for 60 s in the pool in which the platform had been removed. The percentage of time spent in each quadrant was recorded.

### Immunohistochemical analysis

After the MWM test, mice were given an intraperitoneal injection of sodium pentobarbital (> 120 mg/kg). After confirming the animal’s death by the absence of breathing and cardiac arrest, all blood was collected. Then cardiac perfusion was performed with 0.1 M PBS, the brains were removed from the skull, and the brain was separated into the right and left hemispheres with a scalpel. The left hemisphere was fixed in 4% paraformaldehyde for 24 h and then immersed in 15% sucrose solution. The right hemisphere was immediately frozen in liquid nitrogen and stored at − 80 °C until miRNA and RNA extraction. The left hemispheres were frozen and cut in the sagittal plane in 20 μm sections, and then three sections that included the hippocampal area and CP (1.4–2.4 mm lateral from the bregma) were used for immunohistochemical staining.

The sections were incubated overnight at 4 °C with primary antibodies (listed in Supplementary Table 1). Secondary antibodies are listed in Supplementary Table 2. DAPI (Dojindo, Kumamoto, Japan) was used for nuclear staining. Confocal laser scanning microscopy (Nikon A1; Nikon, Tokyo, Japan) was used to obtain the images.

The average Aβ-positive area in three fields of 200 × 200 µm, the average number of NeuN-positive cells in two fields of 150 × 150 µm, and the average intensity of synaptophysin (expressed in arbitrary unit) in three fields of 200 × 200 µm were analyzed in the subiculum area per section. The number of GFAP-positive cells in one field of 310 × 310 µm, the average GFAP-positive area in three fields of 150 × 150 µm, the average TNFα-positive area co-localized with GFAP in three fields of 200 × 200 µm, and the number of Iba1-positive cells in one field of 310 × 310 µm were analyzed in the subiculum area per section. Resting microglia were identified by smaller cell bodies (area < 70 µm^2^) with long branching processes, and activated microglia were identified by larger cell bodies (area > 70 µm^2^) with decreased ramification of branches. The M1 type of activated microglia was identified as MHC class II-positive activated microglia, and the number of MHC class II-negative activated microglia was also counted. The density of F4/80-positive cells in the CP area (10^4^ µm^2^) and the intensity of TTR (expressed in arbitrary unit) in the CP area were analyzed per section. For quantitative analysis, we used Nikon NIS Elements AR software.

After PKH-labeled BM-MSCs were injected, mice were sacrificed as described above. The right hemispheres were immersed in 4% paraformaldehyde for 24 h and then placed into 15% sucrose solution. The right hemispheres were sagittally cut into 20-µm thick frozen sections, and six selected sections that included the lateral ventricle (0.9–2.4 mm lateral from the bregma) and that were 240 µm apart were chosen. The number of PKH-positive cells that attached to the CP was counted per section.

### Isolation of exosomes from the CM of BM-MSCs and labeling with PKH

When BM-MSCs reached 90% confluency after three passages in 15-cm dishes (TPP Techno Plastic Products AG, Trasadingen, Switzerland), the cells were washed with PBS, and the conventional medium was replaced with 10 ml FBS-free alpha-minimal essential medium. After incubation for 24 h, the total culture medium was collected. MagCapture Exosome Isolation Kit PS (FUJIFILM Wako Pure Chemical Corp, Osaka, Japan) was used to isolate exosomes from the medium, according to the manufacturer’s protocol. Exosomes (0.24 µg) were then labeled with PKH (Sigma-Aldrich), according to the manufacturer’s protocol. As a control, exosomes isolated from PBS using MagCapture Exosome Isolation Kit PS were labeled with PKH.

### Adding PKH-labeled exosomes to astrocytes

On day 1 after seeding astrocytes in poly-l-lysine (Sigma-Aldrich)-coated four-well chamber slides (Sigma-Aldrich), BM-MSC-derived PKH-labeled exosomes (0.24 µg) were suspended in 500 µl medium (DMEM/F-12 (Thermo Fisher Scientific) with 10% FBS and 1% PS). PBS-derived PKH-labeled exosomes were also suspended in 500 µl medium. Then, medium containing PKH-labeled exosomes was added to one well of a four-well chamber slide.

At 24 h after adding the medium, the chamber was washed with PBS, and cells were fixed with 4% paraformaldehyde for 2 h. After permeabilization with PBS with Tween 20 (0.05%), the cells were incubated with antibodies for GFAP at room temperature for 2 h (Suppl. Table 1). After washing, the cells were incubated with a FITC-conjugated secondary antibody for 1 h at room temperature (Suppl. Table 2). DAPI was used for nuclear staining. Images were obtained with a confocal laser scanning microscope (Nikon A1).

### Co-culture of astrocytes with BM-MSCs using the transwell culture system

BM-MSCs after three passages were seeded onto a transwell insert (0.4-µm porous membrane) for 24-well plates (Corning, Corning, NY, USA) at a density of 1.0 × 10^5^ cells/cm^2^. At 24 h after seeding, BM-MSCs were transfected with 10 nM miR-146a mimic (QIAGEN, Hilden, Germany) using Hiperfect transfection reagent (QIAGEN). At 4 h after transfection, BM-MSCs in the insert and astrocytes that were seeded at 5.0 × 10^4^ cells/cm^2^ in 24-well plates 3 days before were combined to start the co-culture. The conventional medium was replaced with DMEM/F-12 medium containing 10% exosome-depleted FBS (EXO-FBS-50 A-1, System Biosciences, Palo Alto, CA, USA) and 1% PS. Non-transfected BM-MSCs in the insert were also co-cultured with astrocytes in the same way. After 72 h of co-culture, astrocytes were collected by trypsinization. Both miRNA and mRNA of astrocytes were extracted with a mirVana miRNA isolation kit (Thermo Fisher Scientific). The TaqMan MicroRNA Assay protocol (Thermo Fisher Scientific) was used to synthesize cDNA from targeted miRNA. mRNA was converted into cDNA using the Omniscript RT Kit (QIAGEN). Real-time PCR was performed by using TaqMan Universal Master Mix II (Thermo Fisher Scientific) or SYBR green (Thermo Fisher Scientific) on an Applied Biosystems 7,500 apparatus (Thermo Fisher Scientific). The primers for miRNA and mRNA are listed in Supplementary Tables 3 and 5, respectively. Using snoRNA135 as an endogenous control, relative expression of miR-146a was calculated with the 2^−ΔΔCt^ comparative method. Using GAPDH as an endogenous control, relative expression of mRNA (IRAK1, TRAF6, and NF-κB) was calculated with the 2^−ΔΔCt^ comparative method.

### Adding exosomes derived from miR-146a-transfected BM-MSCs into astrocytes

BM-MSCs of passage three were used for culturing. BM-MSCs were seeded at a density of 1.5 × 10^4^ cells/cm^2^ in a 15-cm dish. BM-MSCs were cultured in alpha-minimal essential medium with 15% FBS and 1% PS. When BM-MSCs reached 90% confluence, the dish was transfected with 10 nM miR-146a using Hiperfect transfection reagent. At 4 h after transfection, the dish was washed with PBS, and the conventional medium was replaced with 10 ml FBS-free alpha-minimal essential medium. After 24 h, the medium was collected, and exosomes were isolated with a MagCapture Exosome Isolation Kit PS. On day 1 after seeding astrocytes in poly-l-lysine-coated 96-well plates (Corning) at a density of 3.1 × 10^4^/cm^2^, exosomes suspended in FBS-free DMEM/F-12 medium were added to astrocytes (0.4 µg/ml). After 24 h of adding exosomes, both miRNA and mRNA of astrocytes were extracted as described above. cDNA synthesis and real-time PCR were also performed as described above.

### Statistical analysis

Data are expressed as the means ± standard error of the mean (SEM). Statistical analysis was performed using the two-tailed unpaired *t*-test, one-way analysis of variance (ANOVA) for repeated measures, or one-way ANOVA followed by the Tukey test for post-hoc comparisons^45,46^. When two factors were assessed, two-way ANOVA with the Tukey test was carried out^45^. GraphPad Prism (GraphPad Software Inc., San Diego, CA, USA) was used for statistical analysis, and differences were considered significant at *P* < 0.05^45^.

## Supplementary information

Supplementary file1 (PDF 500 kb)
