# Supplementary material for: Bone marrow-derived mesenchymal stem cells improve cognitive impairment in an Alzheimer’s disease model by increasing the expression of microRNA-146a in hippocampus
Source: Sci Rep. 2020 Jul 1;10:10772. doi: 10.1038/s41598-020-67460-1 (PMC7330036; doi:10.1038/s41598-020-67460-1)
Supplement: Supplementary file 1 — Supplementary file1 (PDF 500 kb) [file 41598_2020_67460_MOESM1_ESM.pdf]

Supplementary information

Bone marrow-derived mesenchymal stem cells improve cognitive impairment in an Alzheimer's disease model by increasing the expression of microRNA-146a in hippocampus

Masako Nakano[1]*, Kenta Kubota[1,2], Eiji Kobayashi[1,3], Takako S. Chikenji[1,4], Yuki Saito[1], Naoto Konari[1], Mineko Fujimiya[1]

1 Department of Anatomy, Sapporo Medical University School of Medicine, Sapporo, Hokkaido, Japan
2 Department of Physical Therapy, Hokkaido Chitose Rehabilitation College, Chitose, Hokkaido, Japan
3 Graduate School of Rehabilitation Science, Hokkaido Bunkyo University, Eniwa, Hokkaido, Japan
4 Department of Health Sciences, School of Medicine, Hokkaido University, Sapporo, Hokkaido Japan

*Corresponding author

Supplementary information

1. Supplementary Figures
2. Supplementary Tables
3. Methods
4. References

Supplementary Figure 1

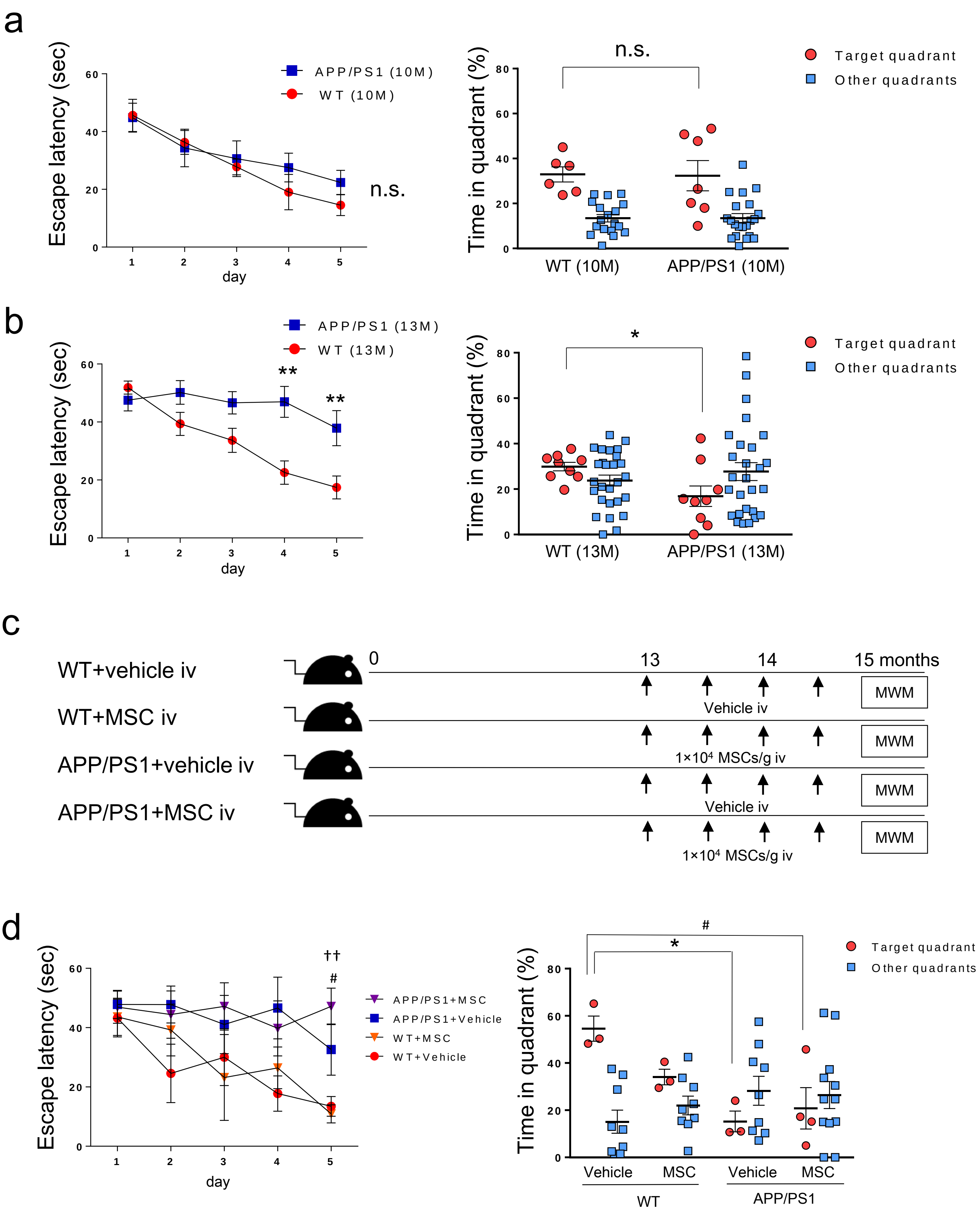

Supplementary Figure 1. The MWM test of APP/PS1 mice at 10 and 13 months of age and the effects of intravenously injected BM-MSCs in APP/PS1 mice. (a) The hidden platform test of the MWM test of APP/PS1 mice at 10 months of age (left panel). n = 6-7/group. Values are the means of four trials per day ± SEM. Two-way ANOVA, for repeated measures, Bonferroni post-hoc test. The probe test of the MWM test of APP/PS1 mice at 10 months of age (right panel). n = 6-7/group. Values are the means ± SEM. Two-tailed unpaired *t*-test. (b) The hidden platform test of the MWM test of APP/PS1 mice at 13 months of age (left panel). n = 9/group. Values are the means of four trials per day ± SEM. **$P < 0.01$, two-way ANOVA, for repeated measures, Bonferroni post-hoc test. The probe test of the MWM test of APP/PS1 mice at 13 months of age (right panel). n = 9/group. Values are the means ± SEM. *$P < 0.05$, two-tailed unpaired *t*-test. (c) Experimental protocol. At 13 months old, WT and APP/PS1 mice were injected intravenously with 1×10^4 BM-MSCs/g body weight or vehicle four times at 2-week intervals. (d) The hidden platform test of the MWM test (left panel). n = 3-4/group. Values are the means of four trials per day ± SEM. #$P < 0.05$, WT+vehicle vs. APP/PS1+MSC; ††$P < 0.01$, WT+MSC vs. APP/PS1+MSC. Two-way ANOVA, Tukey post-hoc test, at each day. The probe test of the MWM test (right panel). n = 3-4/group. Values are the means ± SEM. *$P < 0.05$, WT+vehicle vs. APP/PS1+vehicle; #$P < 0.05$, WT+vehicle vs. APP/PS1+MSC, two-way ANOVA, Tukey post-hoc test.

Supplementary Figure 2

a

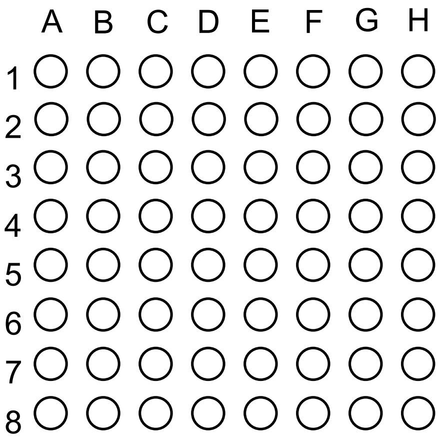

A1,A2	Positive Control
B1,B2	Positive Control
C1,C2	Negative Control
D1,D2	Negative Control
E1,E2	CINC-2
F1,F2	CINC-3
G1,G2	CNTF
H1,H2	CX3CL1
A3, A4	GM-CSF
B3, B4	IFN-gamma
C3, C4	IL-1α
D3, D4	IL-1β
E3, E4	IL-4
F3, F4	IL-6
G3, G4	IL-10
H3,H4	CXCL5

A5, A6	Leptin
B5, B6	MCP-1
C5, C6	MIP-3α
D5, D6	β-NGF
E5, E6	TIMP-1
F5, F6	TNFα
G5, G6	VEGF-A
H5, H6	Blank
A7, A8	Blank
B7, B8	Blank
C7, C8	Blank
D7, D8	Blank
E7, E8	Blank
F7, F8	Blank
G7, G8	Blank
H7, H8	Positive Control

Supplementary Figure 2. Rat cytokine array. (a) The rat cytokine array coordinates and the names of the cytokines.

Supplementary Figure 3

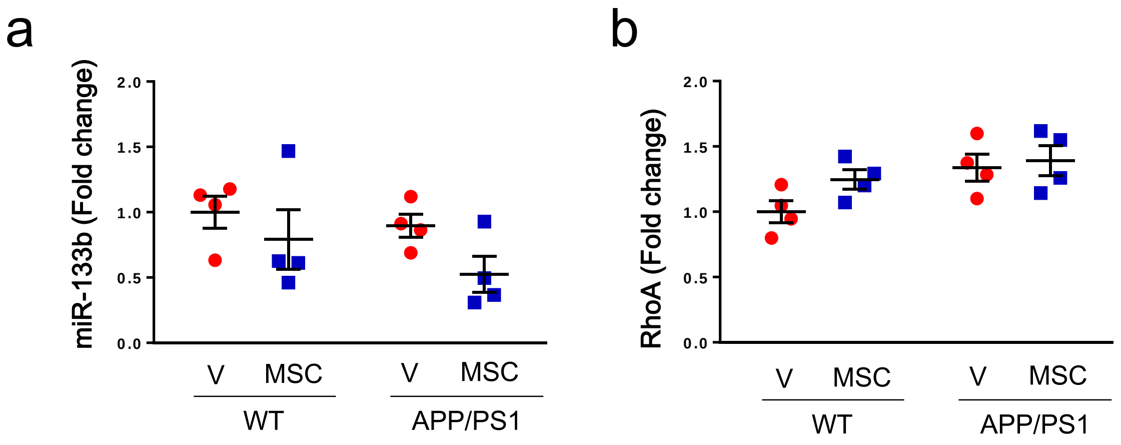

Supplementary Figure 3. Analysis of miRNA and mRNA in the hippocampus. (a) The expression of miR-133b in vehicle- or BM-MSC-treated mice. n = 3-4/group. Values are the means ± SEM. Two-way ANOVA, Tukey post-hoc test. (b) The expression of RhoA in vehicle- or BM-MSC-treated mice. n = 3-4/group. Values are the means ± SEM. Two-way ANOVA, Tukey post-hoc test.

Supplementary Figure 4

a

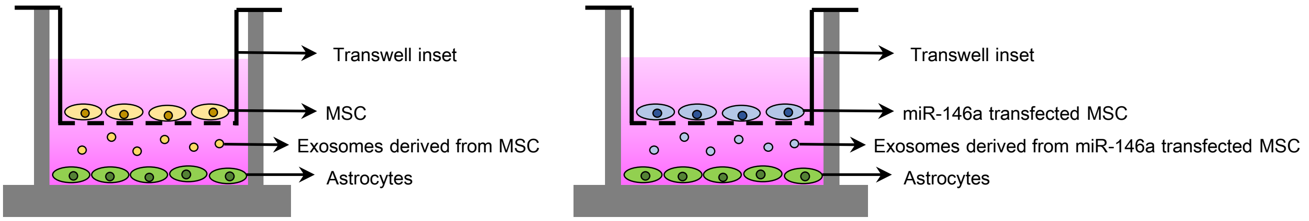

b

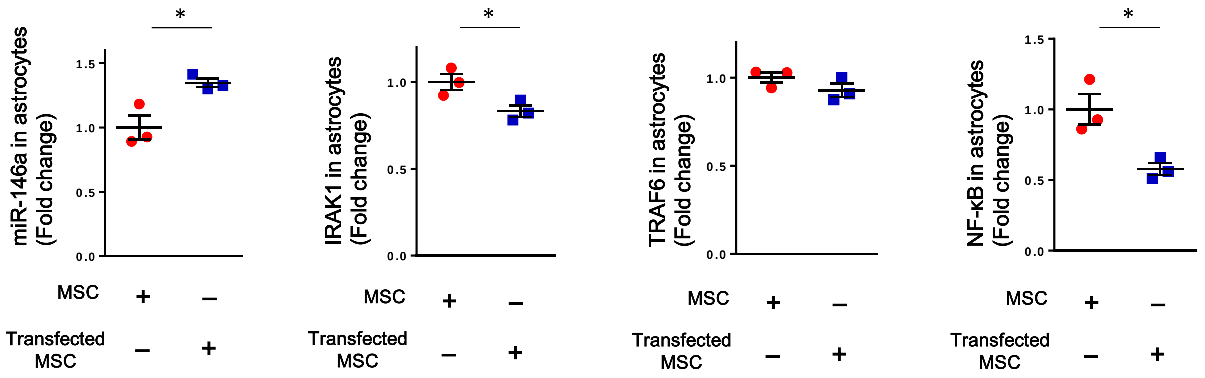

Supplementary Figure 4. Analysis of the effect of BM-MSC-derived exosomal miR-146a. (a) A transwell co-culture assay of astrocytes (bottom well) with BM-MSCs or miR-146a-transfected BM-MSCs (top well). To inhibit cell-cell contact, a 0.4-µm porous membrane was used as an insert. (b) The expression of miR-146a, IRAK1, TRAF6, and NF-κB in astrocytes with BM-MSCs or transfected BM-MSCs. n = 3/group. Values are the means ± SEM. $^*P < 0.05$, two-tailed unpaired t-test.

Supplementary Table 1

Immunofluorescence

Primary Antibody	Source	Dilution	Manufacture
Aβ	rabbit monoclonal	1:500	Cell Signaling
NeuN	rabbit polyclonal	1:1000	Millipore
Synaptophysin	rabbit polyclonal	1:500	Sigma-Aldrich
GFAP	chiken polyclonal	1:500	Millipore
TNFα	rabbit polyclonal	1:500	abcam
Iba1	rabbit polyclonal	1:500	WAKO
MHC class II	rat monoclonal	1:200	Santa Cruz Biotechnology
F4/80	rat monoclonal	1:500	Santa Cruz Biotechnology
TTR	sheep monoclonal	1:500	abcam

Supplementary Table 1. Primary antibodies used for immunofluorescence

Supplementary Table 2

Immunofluorescence

Second Antibody	Conjugate	Dilution	Manufacture
Rabbit IgG	FITC	1:500	Millipore
Rabbit IgG	Cy3	1:500	Jackson ImmunoResearch
Chicken IgG	FITC	1:500	Millipore
Rat IgG	Alexa Fluor 488	1:500	Jackson ImmunoResearch
Rat IgG	Alexa Fluor 647	1:500	Jackson ImmunoResearch
Sheep IgG	FITC	1:500	Jackson ImmunoResarch

Supplementary Table 2. Secondary antibodies used for immunofluorescence

Supplementary Table 3

PCR primers for miRNA analysis

Gene	Sequence
miRNA-146a	5’-UGAGAACUGAAUUCCAUGGGUU-3’
miRNA-133b	5’-UUUGGUCCCCUUCAACCAGCUA-3’
snoRNA135	5’-CUAAAAUAGCUGGAAUUACCGGCAGAUUGGUAGUGGUGAGCCUAUGGUUUUCUGAAG-3’
cel-miRNA-39	5’-UCACCGGGUGUAAAUCAGCUUG-3’

Supplementary Table 3. PCR primers for miRNA analysis

Supplementary Table 4

mRNA PCR primers for mouse hippocampus

Gene	Direction	Sequence
IRAK1	forward	5'-CCACCCTGGGTTATGTGCC-3'
	reverse	5'-CCACCCTGGGTTATGTGCC-3'
TRAF6	forward	5'-CCACCCTGGGTTATGTGCC-3'
	reverse	5'-GAGGATGTGAACGAGGTCAGC-3'
NF-κB	forward	5'-GGAGGCATGTTCGGTAGTGG-3'
	reverse	5'-CCCTGCGTTGGATTTCGTG-3'
RhoA	forward	5'-AGCTTGTGGTAAGACATGCTTG-3'
	reverse	5'-GTGTCCCATAAAGCCAACTCTAC-3'
GAPDH	forward	5'-ACGACCCCTTCATTGACC-3'
	reverse	5'-CCAGTGAGCTTCCCGTTCAGC-3'

Supplementary Table 4. PCR primers for mRNA analysis of mouse hippocampus

Supplementary Table 5

mRNA PCR primers for rat cultured astrocytes

Gene	Direction	Sequence
IRAK1	forward	5’-GCTGTGGACACCGAT-3’
	reverse	5’-GCTACACCCATCCACA-3’
TRAF6	forward	5’-CAGTCCCCTGCACATT-3’
	reverse	5’-GAGGAGGCATCGCAT-3’
NF-κB	forward	5’-AATTGCCCCGGCAT-3’
	reverse	5’-TCCCGTAACCGCGTA-3’
GAPDH	forward	5’-ACCACAGTCCATGCCATCAC-3’
	reverse	5’-TCCACCACCCTGTTGCTGTA-3’

Supplementary Table 5. PCR primers for mRNA analysis of rat cultured astrocytes

Supplementary Methods

Intravenous injection of BM-MSCs.

At 13 months of age, female APP/PS1 and WT mice were injected with BM-MSCs (1 × 10^4 BM-MSCs/g body weight per mouse in 200 μl phosphate-buffered saline (PBS)) or vehicle (200 μl PBS) into the tail vein (Suppl. Fig. 1c). After intravenous injection four times at 2-week intervals, the MWM test was conducted.

Electron microscopic observations.

Electron microscopic observations were done as described previously[1)] . In brief, the left hemispheres were cut into 100-μm thick sagittal sections and fixed with 2.5% glutaraldehyde (WAKO, Osaka, Japan). Then, sections were post-fixed with 2% osmium tetroxide and embedded in an epoxy resin (EPON812, TAAB Laboratories Equipment, Berks, UK). Sections were cut at 70 nm thick and stained with uranyl acetate and lead citrate. Then, the subiculum area was observed with a transmission electron microscope (H7650, HITACHI, Tokyo, Japan) at 30,000× magnification. The synapses were identified by the presence of a post-synaptic density. The average of the number of synapses that were counted in 18 randomly selected fields for each mouse was analyzed.

Rat cytokine antibody array.

BM-MSCs were cultured until 90-100% confluent in 15-cm dishes in alpha-minimal essential medium with 15% FBS and 1% PS. Then, the conventional medium was changed to FBS-free alpha-minimal essential medium with 1% PS.

After 24 h, the media were collected and centrifuged at 2600 ×g for 10 min at 4°C. The supernatant was loaded onto a Rat Cytokine Antibody Array (RayBiotech Life, Norcross, GA, USA), according to the manufacturer's protocol. Digital images were obtained with the Las-3000 imaging system (Fujifilm, Tokyo, Japan).

miRNA and mRNA isolation from the hippocampus and quantitation.

The mirVana miRNA isolation kit was used to extract miRNA from the frozen right hippocampus. The TaqMan MicroRNA Assay protocol was used to synthesize cDNA from targeted miRNA. The primers that targeted miR-146a, miR-133b, and snoRNA 135 are listed in Supplementary Table 3. Applied Biosystems 7500 and TaqMan Universal Master Mix II were used for real-time PCR. Using snoRNA 135 as an endogenous control, relative expression of miR-146a as well as miR-133b was calculated with the $2^{-\Delta\Delta Ct}$ comparative method.

Subicular mRNA from APP/PS1 and WT mice that were injected with BM-MSCs or vehicle was isolated from 4% paraformaldehyde-fixed 100-μm thick sections. The subiculum area was cut out from the sections with a scalpel, and mRNA was isolated using the RecoverAll Total Nucleic Acid Isolation Kit (Thermo Fisher Scientific). Then, mRNA was converted into cDNA using the Sensiscript RT Kit (QIAGEN). Real-time PCR was performed using SYBR green and Applied Biosystems 7500. Using GAPDH as an endogenous control, relative expression of mRNA was calculated with the $2^{-\Delta\Delta Ct}$ comparative method. The primers for IRAK1, TRAF6, NF-κB, RhoA, and GAPDH are listed in Supplementary Table 4.

Isolation and culture of primary rat astrocytes.

Hippocampal tissues of neonatal Sprague Dawley rats (1 or 2 days old) were isolated after inhalation of excess isoflurane, as described previously[2)]. Briefly, cells of hippocampal tissues were cultured in DMEM/F-12 with 10% FBS and 1% PS in poly-L-lysine-coated dishes. After cells of hippocampal tissues were seeded at a density of 1.5×10^5 cells/cm^2, the medium was changed twice weekly. At 7-8 days after seeding, the culture dishes were shaken at 200 rpm overnight to remove unattached cells. Then, attached cells were trypsinized and seeded at 5.0×10^4 cells/cm^2 in poly-L-lysine-coated 24-well plates or at 1.2×10^4 cells/cm^2 in poly-L-lysine-coated four-well chambers. More than 95% of cultured cells were confirmed to be GFAP-positive astrocytes.

Isolation and quantitation of both exosomal miRNA and free-floating miRNA from the CM of BM-MSCs or that of miR-146a-transfected BM-MSCs.

BM-MSCs of passage three were used for culturing. BM-MSCs were seeded at a density of 1.5×10^4 cells/cm^2 in two 15-cm dishes. BM-MSCs were cultured in alpha-minimal essential medium with 15% FBS and 1% PS. When BM-MSCs reached 90% confluence, one dish was transfected with 10 nM miR-146a using Hiperfect transfection reagent, and the other dish was not transfected. At 4 h after transfection, each dish was washed with PBS, and the conventional medium was replaced with 10 ml FBS-free alpha-minimal essential medium. After 24 h, the medium was collected, and exosomes were isolated. ExoQuick-TC (System Biosciences) was used to isolate exosomes, according to the manufacturer’s protocol. After isolation of exosomes, the residual supernatant was used for the analysis of free-floating miRNA.

Using 2 nM exogenous synthetic cel-miR-39 as an external control, the mirVana PARIS Kit (Thermo Fisher Scientific) was used for the extraction of miRNA. For reverse transcription, the TaqMan Advanced miRNA cDNA Synthesis kit (Thermo Fisher Scientific) was used. Real-time PCR was performed using the TaqMan Fast Advanced Master Mix (Thermo Fisher Scientific) and Applied Biosystems 7500. The primers that targeted miR-146a and cel-miR-39 are listed in Supplementary Table 3. Using cel-miR-39 as an external control, relative expression of miR-146a was calculated with the $2^{-\Delta\Delta Ct}$ comparative method.

Adding miR-146a mimic to astrocytes.

At 24 h after astrocytes were seeded at 5.0×10^4 cells/cm^2 in poly-L-lysine-coated 24-well plates, 10 nM miR-146a mimic without Hiperfect transfection reagent was added to the medium to examine whether free-floating miR-146a affected the expression of miR-146a in astrocytes. At 24 h after adding the miR-146a mimic, astrocytes were collected by trypsinization. The miRNA of astrocytes was extracted with a mirVana miRNA isolation kit. cDNA synthesis and real-time PCR were performed as described above. The primers for miRNA are listed in Supplementary Table 3. Using snoRNA135 as an endogenous control, relative expression of miRNA-146a was calculated with the $2^{-\Delta\Delta Ct}$ comparative method.

Supplementary References

1) Nakano, M. et al. Bone marrow-derived mesenchymal stem cells improve diabetes-induced cognitive impairment by exosome transfer into damaged neurons and astrocytes. *Sci Rep* 6, 24805 (2016).
2) Kubota, K. et al. An enriched environment prevents diabetes-induced cognitive impairment in rats by enhancing exosomal miR-146a secretion from endogenous bone marrow-derived mesenchymal stem cells. *PLoS One* 13, e0204252 (2018).
